# Compartmentalized Regulation of Pulmonary and Systemic Inflammation in Critical COVID-19 Patients

**DOI:** 10.3390/v15081704

**Published:** 2023-08-08

**Authors:** Luciana Santiago, Marcela Helena Gonçalves-Pereira, Mariana Sousa Vieira, Cecilia Gómez Ravetti, Paula Frizera Vassallo, Rafael Silva e Castro, Pedro Pires Costa Pimenta, Marcus Vinícius Melo de Andrade, Helton da Costa Santiago, Vandack Nobre

**Affiliations:** 1 Faculdade de Medicina, Universidade Federal de Minas Gerais, Belo Horizonte 31270-901, MG, Brazil; lsantiagobr@gmail.com (L.S.); ceciliag.ravetti@gmail.com (C.G.R.); castro.rafael8991@gmail.com (R.S.e.C.); pedropcpimenta@gmail.com (P.P.C.P.); andradem@ufmg.br (M.V.M.d.A.); 2 Hospital das Clínicas, Universidade Federal de Minas Gerais, Belo Horizonte 30130-100, MG, Brazil; pfvassallo1@gmail.com; 3 Departamento de Bioquímica e Imunologia, Instituto de Ciências Biológicas, Universidade Federal de Minas Gerais, Belo Horizonte 31270-901, MG, Brazil; marcelahgpo@gmail.com (M.H.G.-P.); vieira.msousa@gmail.com (M.S.V.); 4 Núcleo Interdisciplinar de Investigação em Medicina Intensiva (NIIMI), Belo Horizonte 31270-901, MG, Brazil

**Keywords:** COVID-19, SARS-CoV-2, cytokines, chemokines, lung, blood, inflammation

## Abstract

Critical COVID-19 has been associated with altered patterns of cytokines. Distinct inflammatory processes in systemic and pulmonary sites have been reported, but studies comparing these two sites are still scarce. We aimed to evaluate the profile of pulmonary and systemic cytokines and chemokines in critically ill COVID-19 patients. Levels of cytokines and chemokines were measured in plasma samples and minibronchoalveolar lavage of critical COVID-19 patients within 48 h and 5–8 days after intubation. Distinct inflammatory processes were observed in the lungs and blood, which were regulated separately. Survivor patients showed higher lung cytokine levels including IFN-γ, IL-2, IL-4, G-CSF, and CCL4, while nonsurvivors displayed higher levels in the blood, which included IL-6, CXCL8, CXCL10, CCL2, and CCL4. Furthermore, our findings indicate that high TNF and CXCL8 levels in the mini-BAL were associated with better lung oxygen exchange capacity, whereas high levels of IFN-γ in plasma were associated with worse lung function, as measured using the PaO_2_/FiO_2_ ratio. These results suggest that a robust and localized inflammatory response in the lungs is protective and associated with survival, whereas a systemic inflammatory response is detrimental and associated with mortality in critical COVID-19.

## 1. Introduction

Since the emergence of coronavirus disease 2019 (COVID-19), caused by severe respiratory acute syndrome coronavirus 2 (SARS-CoV-2), researchers from different countries have struggled to understand this new disease and provide guidance for its clinical management. Over the three years since the start of the pandemic began, there have been over 676 million reported cases of COVID-19 and more than 6.8 million deaths worldwide [1].

The pulmonary system is the primary site of SARS-CoV-2, making it a critical player in the initial immune response against this virus [2]. However, COVID-19 is now known to be a systemic disease, with the involvement of multiple extrapulmonary tissues like neurologic, cardiac, vascular, renal, gastrointestinal, and other systems [2,3]. Thus, studying the immune response in both pulmonary and systemic sites is essential for a better understanding of COVID-19 pathogenesis.

A higher incidence of critical illness and fatality has been associated with altered cytokine patterns in patients with COVID-19 [4,5,6]. For example, a longitudinal study demonstrated that while moderate COVID-19 cases had reduced plasma cytokine levels, severe COVID-19 cases maintained high levels of cytokines and chemokines such as Il-2, Il-16, CCL1, CCL2, and M-CSF, which were associated with monocytes and T cell recruitment and activation, and survival [7]. Another study found that severe cases admitted to the intensive care unit (ICU) had increased plasma levels of IL-2, IL-7, IL-10, G-CSF, CXCL10, CCL2, CCL3, and TNF when compared to those with mild or moderate COVID-19 [8]. Furthermore, elevated plasma levels of cytokine IL-6 have been shown to be a predictor of lung injury severity [9] and mortality [10] in COVID-19. Additionally, cytokine levels are also elevated in the lungs during SARS-CoV2 infection. For instance, analysis of transcriptomic data in bronchoalveolar lavage of a patient with COVID-19 found high expression of CCL2, CCL3, CCL4, and CXCL10 when compared to healthy donors [11].

Some studies have attempted to understand the regulation of systemic and pulmonary inflammation during COVID-19. For example, a positive correlation was found between lungs and peripheral inflammation in relation to IL-6 (r = 0.53, *p* < 0.01) [12] and CXCL-10 (r^2^ = 0.99, *p* < 0.0001) [13] levels in COVID-19. However, another report suggests limited correlations between the inflammatory cytokines in these two sites [14]. Therefore, further studies are still necessary to understand how local and systemic inflammations are regulated in COVID-19.

In this study, our objective was to assess the cytokine patterns in paired samples obtained from the pulmonary and systemic sites of patients with critical COVID-19 who required invasive mechanical ventilation. To achieve this, we analyzed the levels and correlations of cytokine in minibronchoalveolar lavage (mini-BAL) fluid and plasma at two different time points. Additionally, we examined the relationship between cytokine levels from the pulmonary and systemic sites and laboratory and clinical parameters, as well as the association of these proteins with the disease outcome of patients with critical COVID-19.

## 2. Materials and Methods

### 2.1. Patients and Ethical Aspects

This prospective cohort study was approved by the Research Ethics Committee of the Universidade Federal de Minas Gerais, (CAAE: 31336920.5.0000.5149), and informed consent was obtained from all participants or their legal representatives. Clinical data, available in the patients’ chart or from patients or their relative’s information, and biological samples were collected from patients who were admitted to the intensive care unit (ICU) at Hospital das Clínicas of the Federal University of Minas Gerais, Belo Horizonte, Brazil, from May 2020 to April 2021. Adult patients aged 18 years or older with COVID-19 and respiratory failure who were on invasive mechanical ventilation (MV) were enrolled. Co-morbidities were classified according to ICD-10. Samples were collected on two time points: within the first 48 h of intubation (D1–2) and around day 7 (D5–8) after intubation. Patients were followed up until the outcome (death or hospital discharge) and were later divided into two groups for analysis: survivors and nonsurvivors. Additional patients were enrolled to replace early withdrawals, as some patients died or were extubated before day 7 (see Figure S1 for a detailed of the timepoint for sample collection). The clinical management was exclusively determined by the healthcare team and followed the current guidelines of the hospital and Brazilian Ministry of Health.

### 2.2. Sample Collection and Processing of Biological Material

Peripheral blood and pulmonary secretion samples were collected on the same day at two timepoints: days 1–2 (D1–2) and days 5–8 (D5–8) after intubation. Peripheral blood was collected in heparinized vacuum collection tubes with a volume of 5 mL. Plasma and peripheral blood mononuclear cells (PBMCs) were separated by density gradient after centrifugation using a Ficoll Histopaque-1077 gradient (SIGMA-Aldrich, St. Louis, MO, USA). The plasma was stored in a freezer at −80 °C until analysis of cytokines and chemokines, and cells were used in different studies [15,16].

Pulmonary secretion samples were collected using a modified minibronchoalveolar lavage (mini-BAL) technique [17], using a smaller volume [18], and a closed tracheal aspiration system. Briefly, 10 mL of sterile 0.9% saline solution was instilled into the trachea, recovered, and immediately dispensed into a sterile secretion collection bottle, keeping the system closed during the procedure. The pulmonary secretion was processed within two hours of collection using the method previously described [19], which consists of diluting the pulmonary secretion sample 2x in phosphate buffer saline (PBS 1x) with a pH of 7.4 containing 0.1% dithiothreitol (LGC Biotechnology, Teddington, UK). The sample was then vortexed for 15 s (s), allowed to rest for 10 min (min), and vortexed again for 15 s [19]. Next, the samples were further diluted 4x in PBS 1x and subjected to centrifugation at 200× *g* for 10 min. Finally, the supernatant was collected and stored in a freezer at −80 °C for further analysis of cytokines and chemokines, while the cells were used for different experiments [15,16].

### 2.3. Measurement of Cytokine and Chemokine

The levels of CCL2, CCL4, CXCL8, IL-1β, IL-2, IL-4, IL-5, IL-6, IL-7, IL-10, IL-12, IL-13, IL-17, G-CSF, GM-CSF, TNF, and IFN-γ were measured using the Bio-Plex Pro™ Human Cytokine 17-plex Assay (Bio-Rad, Hercules, CA, USA), while CCL3, CCL5, and CXCL-10 were measured using the Bio-Plex Pro™ Human Cytokine 3-plex Assay (Bio-Rad), following the manufacturer’s instructions. Sample analysis was performed using the Bio-Plex™ 200 System (Bio-Rad) and Bio-Plex Manager™ software version 6.1 (Bio-Rad). However, IL-7 was not detectable, and CCL5 was detectable in only 42% of the total samples (or in only 31% of the pulmonary secretion supernatant); therefore, they were excluded from the analysis.

### 2.4. Statistical Analysis

The statistical analysis was performed using the GraphPad Prisma software version 9.5.0. The data were described in the form of proportion, central tendency, and distribution. The Fisher exact test was used to compare qualitative variables, while the Mann–Whitney test was used for continuous variables. Correlation analysis was conducted using the nonparametric Spearman R test. Outliers were analyzed using the ROUT method (Q = 1%) available in the GraphPad Prisma software version 9.5.0. A value of *p* < 0.05 was considered statistically significant in all analyses, using a two-tailed test.

## 3. Results

### 3.1. Characteristics of the Study Population

We prospectively enrolled 34 severely ill patients with SARS-CoV-2 infection under invasive mechanical ventilation, who were admitted to the intensive care unit (ICU) of the Hospital das Clínicas of the Federal University of Minas Gerais, Belo Horizonte, Brazil, between May 2020 and April 2021. The median age was 58 years, and 62% of them were male. The most common underlying comorbidities were systemic arterial hypertension (16%), diabetes mellitus (12%), and cardiovascular disease (9%). Paired blood samples and minibronchoalveolar lavage (mini-BAL) were collected at two time points of the followup: the first 48 h after intubation (D1–2) and days 5–8 (D5–8) after intubation. Patients were followed up until the outcome, either hospital discharge (survivors, *n* = 16) or death (nonsurvivors, *n* = 18). Patients in the nonsurvivors group were older than the survivors (*p* = 0.02) and had a significantly higher number of comorbidities (*p* < 0.05), with a predominance of hypertension, diabetes mellitus, and cardiovascular disease. These characteristics are commonly observed in patients who progress to a more severe state in COVID-19 [20]. The main characteristics of the included patients are presented in Table 1.

### 3.2. Compartmentalized Correlation of Cytokines Levels between Lung and Plasma

**Figure 1:**
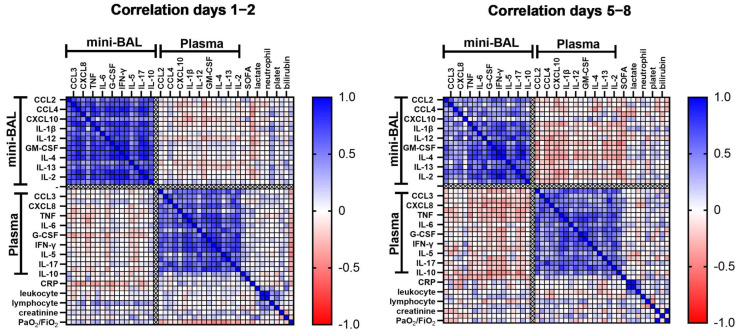
Correlation matrix of cytokine levels and laboratory parameters within and between lung and plasma. Cytokines, chemokines, and laboratory parameters were measured in mini-BAL and peripheral plasma on days 1–2 and days 5–8 after intubation (*n* = 34). Correlations were analyzed using the Spearman’s rank correlation coefficient. Each column represents a single cytokine, and the intensity of the color represents the level of correlation (r) between cytokine pairs. Blue color indicates a positive correlation while red represents a negative correlation. Abbreviations: CRP, C-reactive protein; G-CSF, granulocyte colony-stimulating factor; GM-CSF, granulocyte-macrophage colony-stimulating factor; IFN-γ, interferon γ; IL, interleukin; SOFA, Sequential Organ Failure Assessment; PaO_2_/FiO_2_, arterial oxygen partial pressure/fractional inspired oxygen; TNF, tumor necrosis factor.

To better understand the immune response in both pulmonary and systemic sites, we measured the concentration of 18 cytokines and chemokines in plasma and mini-BAL up to 48 h after intubation, days 1–2 (D1–2), and around day 7 after intubation, and days 5 to 8 (D5–8). It is already established that cytokine levels are upregulated in the lungs [21] and plasma [6,7,10,22] during severe COVID-19. To verify the relationship between these two compartments, we evaluated the correlations of the cytokines within each site and between them. We observed a strong correlation between the levels of several cytokines within each compartment at both time points, suggesting an internal regulation of the inflammatory processes in each site. However, we did not observe any correlation between cytokine levels in the lungs and plasma, suggesting that the cytokines response in the lungs and circulation are independently regulated (Figure 1, Tables S1–S4).

**Figure 2:**
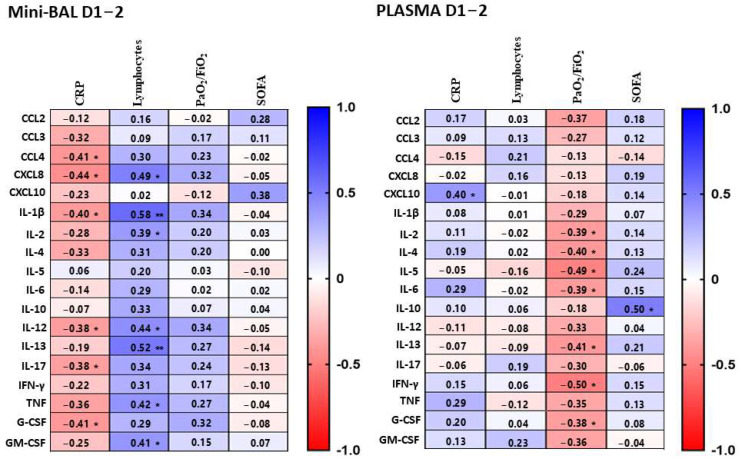
Correlations between cytokines and laboratory data (CRP and lymphocytes), marker of lung injury (PaO_2_/FiO_2_) and clinical severity score (SOFA). Cytokines were measured in mini-BAL and plasma samples on days 1–2 after intubation and were correlated with CPR, lymphocytes, PaO_2_/FiO_2_, and SOFA. Mini-BAL and plasma cytokine levels were correlated to CRP (*n* = 28), lymphocytes count (*n* = 28), PaO_2_/FiO_2_ (*n* = 28), and SOFA (*n* = 28). Correlations were calculated using the Spearman’s rank correlation. Each line represents a single cytokine, and the intensity of the color represents the level of correlation (r). Blue color indicates a positive correlation while red represents a negative correlation. Significant correlations are indicated by * *p* < 0.05 or ** *p* <0.01.

We also analyzed whether any cytokines measured in mini-BAL and plasma could be correlated with organic/laboratory markers, such as lactate, C-reactive protein (CRP), platelets, leukocytes, neutrophils, lymphocytes, creatinine, bilirubin, lung injury (PaO_2_/FiO_2_), and clinical score measured by SOFA at both time points, D1–2, and D5–8 of intubation (Figure 1). We found that, on D1–2, CRP was negatively correlated with pulmonary levels of CCL-4 (r = −0.41, *p* = 0.03), CXCL8 (r = −0.44, *p* = 0.02), IL-1β (r = −0.40, *p* = 0.04), IL-12 (r = −0.38, *p* = 0.04), IL-17 (r = −0.38, *p* = 0.04), and G-CSF (r = −0.41, *p* = 0.03). Surprisingly, in plasma, CRP only showed a positive correlation with CXCL10 (r = 0.40, *p* = 0.04). These results imply that there could be an inverse correlation between pulmonary and systemic inflammation, as suggested by the levels of lung cytokines and CRP. Lymphocytes were also positively correlated with pulmonary levels of CXCL8 (r = 0.49, *p* = 0.01), IL-1β (r = 0.58, *p* < 0.00), IL-2 (r = 0.39, *p* = 0.04), IL-12 (r = 0.44, *p* = 0.02), IL-13 (r = 0.52, *p* < 0.00), TNF (r = 0.42, *p* = 0.03), and GM-CSF (r = 0.41, *p* = 0.03). Interestingly, the SOFA score showed a positive correlation only with plasma levels of IL-10 (r = 0.50, *p* = 0.01), and PaO_2_/FiO_2_ ratio showed a negative correlation with several inflammatory cytokines only in plasma, such as IL-2 (r = −0.39, *p* = 0.04), IL-4 (r = −0.40, *p* = 0.03), IL-5 (r = −0.49, *p* = 0.01), IL-6 (r = −0.39, *p* = 0.04), IL-13 (r = −0.41, *p* = 0.03), IFN-γ (r = −0.50, *p* = 0.01), and G-CSF (r = −0.38, *p* = 0.04) (Figure 2, Tables S1 and S2), which also suggests that systemic inflammation seems correlated with worse clinical and laboratory markers than pulmonary inflammation. It is worth noting that these correlations were mostly lost at D5–8, except for lymphocytes, which maintained a correlation with lung levels of IL-1β (r = 0.42, *p* = 0.03) (Tables S3 and S4). Lactate, leukocytes, neutrophils, and platelets did not correlate with the production of cytokines studied in both sites in this study.

### 3.3. Survivors Exhibit Higher Levels of Cytokines in the Lung, Whereas Nonsurvivors in the Blood

**Figure 3:**
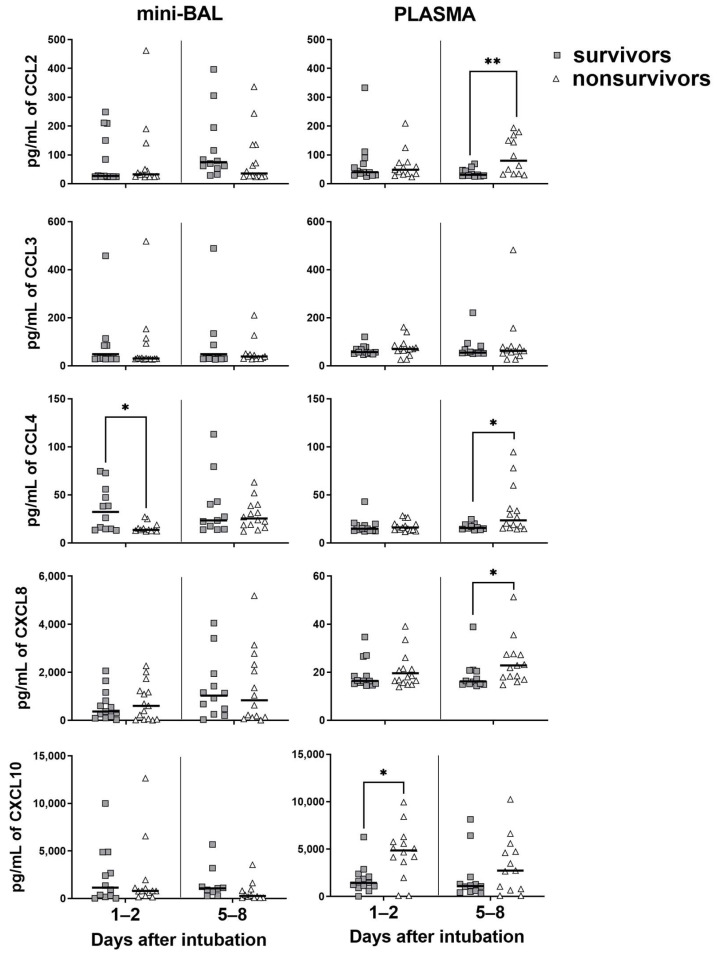
Levels of chemokines in survivor and nonsurvivor group. Chemokines were measured in mini-BAL and plasma up to 48 h on days 1–2 and around day 7 (days 5–8) after intubation. Survivors (gray square): *n* = 13 (days 1–2) and *n* = 12 (days 5–8). Nonsurvivors (open triangle): *n* = 15 (days 1–2) and *n* = 14 (days 5–8). Differences between groups were analyzed using the Mann–Whitney test and are indicated by * when *p* < 0.05 and ** when *p* < 0.01. The black lines represent the median of each group. Outliers were identified using the ROUT method (Q = 1%).

We then analyzed whether cytokines/chemokines measured in plasma or lungs could be associated with the clinical evolution of critical COVID-19. Both groups showed generally similar levels of lung chemokines on D1–2, except for CCL4. The survivors group had a higher production of CCL4 in the mini-BAL on D1–2 (*p* = 0.01) compared to their nonsurvivor counterparts. On the other hand, we observed in the plasma of nonsurvivors a significantly higher production of CXCL10 (*p* = 0.02) on D1–2 and CCL2 (*p* = 0.007), CCL4 (*p* = 0.03), and CXCL8 (*p* = 0.04) on D5–8. We observed no significant difference between the two groups regarding the production of CCL3 in both sites (Figure 3). In contrast, survivors also showed higher levels of IFN-γ (*p* = 0.01), IL-2 (*p* = 0.01), IL-4 (*p* = 0.03), G-CSF (*p* = 0.01), and IL-10 (*p* = 0.02) in mini-BAL on D5–8. On the other hand, nonsurvivors had higher levels of IL-6 in plasma at both time points, D1–2 (*p* = 0.02) and D5–8 (*p* = 0.04). TNF levels were increased in both the lungs and blood on D1–2 in the survival group (Figure 3). No significant differences were found between the two groups in the levels of IL-1β, IL-5 IL-12, IL-13, IL-17, and GM-CSF in either the lungs or blood (Figure S2). These data suggest that nonsurvivors presented higher levels of inflammatory markers in the plasma, especially at latter times of infection, while survivors showed strong inflammatory signs in the lungs.

### 3.4. Systemic Inflammation Is Associated with Worse PaO_2_/FiO_2_ Ratio

Since respiratory failure is a marker of poor evolution of COVID-19, we decided to investigate whether the pulmonary oxygen exchange capacity, as measured by the PaO_2_/FiO_2_ ratio, was associated with either lung or systemic inflammation. The PaO_2_/FiO_2_ ratio is commonly used to evaluate the degree of hypoxemia [23], with a value equal to or below 300 mmHg indicating the presence of acute respiratory distress syndrome according to the Berlin definition [24]. In our study, we chose the cutoff value of 150 mmHg because it indicates worsening of lung function and the need for prone position to improve lung oxygenation capacity in acute respiratory distress syndrome (ARDS) [25].

**Figure 4:**
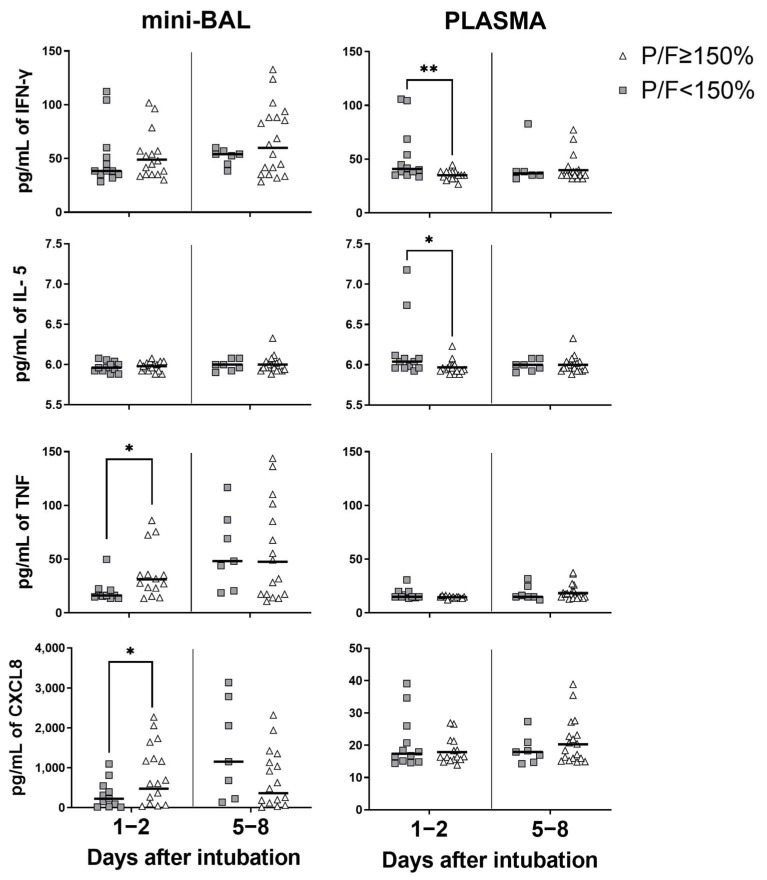
Cytokines/chemokines when PaO_2_/FiO_2_ < 150 mmHg. Cytokines/chemokines measured in minibronchoalveolar lavage (mini-BAL) and plasma when the PaO_2_/FiO_2_ (P/F) < 150 mmHg compared with P/F ≥ 150 mmHg up to 48 h on day 1 (days 1–2) and around day 7 (days 5–8) after intubation. P/F < 150 mmHg (gray square): *n* = 12 (days1–2) and *n* = 7 (days 5–8). P/F ≥ 150 mmHg (open triangle): *n* = 16 (days 1–2) and *n* = 19 (days 5–8). The Mann–Whitney test was used for statistical analysis and was indicated by asterisks (*) when (*p* < 0.05) and (**) when (*p* < 0.01). The black lines represent the median of each group. Outliers were identified using the ROUT test (Q = 1%).

**Figure 5:**
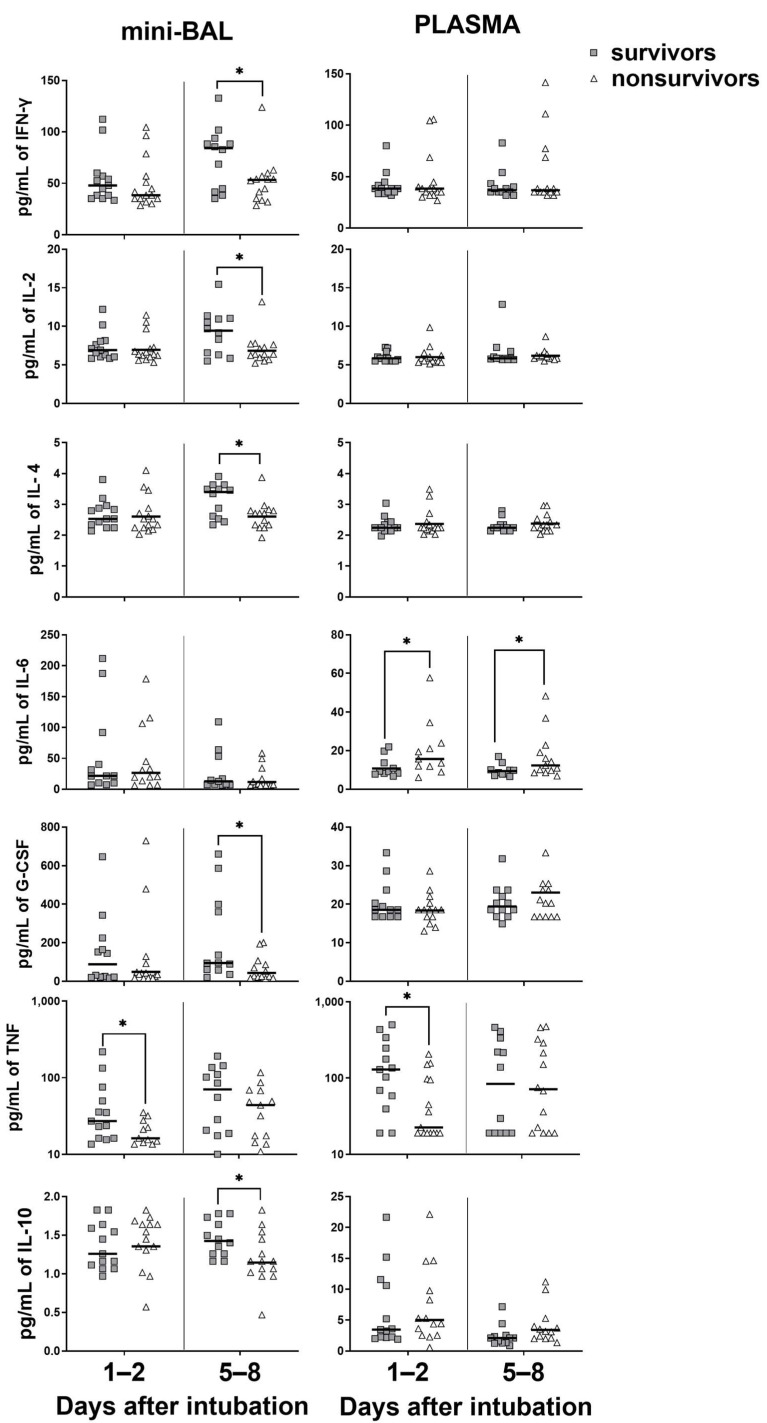
Levels of cytokines in survivor and nonsurvivor group. Cytokines were measured in mini-BAL and plasma up to 48 h on the days 1–2 (days 1–2) and around day 7 (days 5–8) after intubation. Survivors: (gray square) *n* = 13 (days 1–2) and *n* = 12 (days 5–8). Nonsurvivors (open triangle): *n* = 15 (days 1–2) and *n* = 14 (days 5–8). Differences between groups survivors and nonsurvivors on day 1–2 and on day 5–8 were analyzed using the Mann–Whitney test and are indicated by asterisks (*) when statistically significant (*p* < 0.05). The black lines represent the median of each group. Outliers were identified using the ROUT test (Q = 1%).

**Figure 6:**
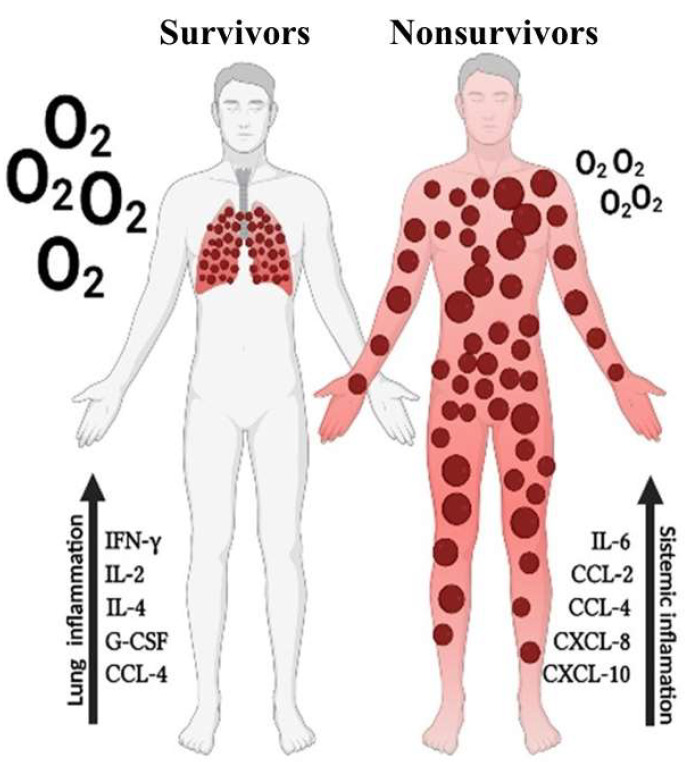
Distinct inflammation patterns in lungs and blood are associated with disease outcome in critical COVID-19. Increased levels of inflammatory markers in the lungs of patients with critical COVID-19 associated with survival while increased levels of inflammatory markers in the peripherical blood associates with death.

We observed patients with a PaO_2_/FiO_2_ ratio equal to or greater than 150 mmHg at point D1–2 of intubation, i.e., patients with better pulmonary function, displayed increased TNF and CXCL8 levels in the lungs (Figure 4). On the other hand, patients with worse pulmonary function with a PaO_2_/FiO_2_ lower than 150 mmHg on D1–2 presented increased levels of inflammatory cytokines such as IFN-γ and IL-5 in the plasma (Figure 5). Notably, these differences were no longer observed on D5–8 of intubation. These findings also suggest that high inflammatory cytokines levels measured in plasma were associated with worse lung function, as measured by the PaO_2_/FiO_2_ ratio at the beginning of the critical phase of infection, while pulmonary inflammation seems to be associated with better lung function (Figure 6).

## 4. Discussion

The study of immune response in critical COVID-19 patients is important for providing information on the pathophysiology of respiratory failure and death associated with SARS-CoV-2 infection, especially in patients who require mechanical ventilation, where mortality ranges from 25% to 34% [26]. There is ample evidence of immune system dysregulation in severe and critical COVID-19 cases [3,6,27,28,29]. However, few studies have focused on the evolution of pulmonary and systemic inflammation in critical patients who recover or die. Although it is established that systemic inflammation can lead to multiorgan dysfunction in critical COVID-19 [6,30,31], the respiratory system is typically the initial site of infection, making it necessary to understand and study inflammation in both sites. Therefore, studying the systemic and pulmonary inflammatory response in critical COVID-19 patients and understanding how these responses occur in the survivors group compared to nonsurvivors is important for comprehending the immunopathogenesis and gaining insights into appropriate treatment management.

Our data show that distinct inflammatory processes are occurring separately in the pulmonary and systemic sites, which significantly impact the infection’s outcome. This finding aligns with the study conducted by Zaid et al., which suggests that plasma cytokines may not reliably indicate inflammation in the lungs of severe COVID-19 patients [14]. Indeed, we found no correlation between lung and blood cytokines levels, but a strong correlation within each site. For instance, when comparing the pulmonary and systemic sites, we observed that the nonsurvivors displayed higher inflammation in the systemic site with high levels of CCL2, CCL4, CXCL8, CXCL10, and IL-6, primarily within days 5–8 after intubation. On the other hand, survivors showed an effective pulmonary inflammation with high levels of mini-BAL TNF and CCL4 within the first 48 h after intubation and exhibited increased levels of IFN-γ, IL-2, IL-4, and G-CSF around 7 days after intubation.

Other studies have also indicated severity associated with increased levels of plasma chemokines, such as CCL2 [6,28,32], CXCL8 [6,32,33], and CXCL10 [6,32,33], in severe COVID-19. The increased presence of chemokines CCL2, CCL4, CXCL8, and CXCL10 in the plasma was observed among the Wuhan, Alpha, Delta, and Omicron variants of moderate to severe COVID-19 patients when compared to healthy individuals. The authors suggest that these chemokines could potentially serve as biomarkers of disease severity or as targets for anti-inflammatory interventions [32]. Increased levels of chemokines in plasma have been associated with an impaired influx of monocytes, neutrophils, and lymphocytes into the lungs. Recent reports have described an enrichment of the alveolar space with macrophages [34,35], T lymphocytes [34], and neutrophils [15,34,35]. On the other hand, the presence of chemokines in the lungs is associated with an increased influx of inflammatory cells [14]. These findings align with our observations that survivors show higher cytokine and chemokine levels in the lungs, possibly allowing an effective inflammation development, whereas nonsurvivors demonstrate elevated levels in the blood, which may impair the migration of inflammatory cells to the lungs. Indeed, decreased levels of CD4+ T cells in the lungs have been associated with failure to extubate and mortality [36]. Likewise, reduced pulmonary neutrophil infiltration is also associated with higher mortality in severe COVID-19 patients over 60 years old [15]. These findings highlight the harmful effects of increased plasma chemokine levels. In our study, elevated systemic concentrations of cytokines and chemokines, along with ineffective pulmonary inflammation, were associated with mortality.

IL-6 has been identified as an important marker of poor COVID-19 evolution [10,28,37,38]. In our study, we also observed a significant increase in IL-6 levels in the plasma of nonsurvivors at both time points, days 1–2 (*p* = 0.02) and days 5–8 (*p* = 0.04) after intubation. This finding supports the use of IL-6 inhibitors as a therapeutic option for critical COVID-19 patients [38,39]. Interestingly, survivors displayed higher levels of TNF in the lungs and plasma during the first 48 h of intubation, which were also associated with higher levels of IL-10. Previous data from our group have shown that balanced proinflammatory and regulatory responses are important for the survival of critical COVID-19 patients [16]. Nonsurvivors, on the other hand, displayed dysfunctional pulmonary and systemic Tregs, which we speculate contribute to harmful inflammation [16].

Indeed, it is interesting to note that the pulmonary oxygen exchange capacity, as indicated by the PaO_2_/FiO_2_ ratio, presented higher correlations with plasma cytokine levels rather than with levels in the lungs. There is data to show a negative correlation of PaO_2_/FiO_2_ with plasma levels of IL-6, G-CSF, CCL2, and CCL3 [28]. In our study, higher plasma levels of IFN-γ and IL-5 were also associated with a PaO_2_/FiO_2_ ratio below 150 mmHg, suggesting that increased peripheral inflammation correlates with a decline in pulmonary oxygen exchange capacity. Even though plasma levels of TNF have been linked to a negative prognosis [10], our data on TNF indicate that both early pulmonary and systemic high TNF levels are associated with favorable outcomes in critical patients. Indeed, not only TNF but also CXCL8 levels in the lungs were associated with a PaO_2_/FiO_2_ ratio above 150 mmHg. This suggests that the presence of an effective inflammatory response in the lungs may be indicative of a better pulmonary oxygen exchange capacity in critically ill patients.

This study provides evidence that inflammation is independently regulated in the lungs and periphery, demonstrating compartmentalization. Furthermore, we found that mortality is associated with dysregulation of the systemic immune response, while an effective balanced pulmonary inflammation is important for the survival of patients with critical COVID-19 (Figure 5). This information has important implications for developing new methods for the management of critical COVID-19, supporting the use of anticytokine and anti-inflammatory therapies, with the goal of reducing the systemic repercussions of uncontrolled inflammation and mortality caused by SARS-CoV-2.

## Figures and Tables

**Table 1:** Main characteristics of the patients included in the study.

	Total	Survivors	Nonsurvivors
	*n* = 34	*n* = 16	*n* = 18
**Baseline**			
Age—median (Q1–Q3)	58 (41–70)	47 * (40–61)	64 * (51–74)
Gender (M)—no. (%)	21 (62)	11 (69)	10 (55)
Symptom days—median (Q1–Q3)	8 (7–11.3)	8 (7–13)	7 (5–11)
SOFA (points)—median (Q1–Q3)	7.5 (5–9)	7 (4–10)	8 (6–9)
APACHE II (points)—median (Q1–Q3)	16.5 (13.5–22)	16 (10.5–21.5)	17.5 (15–23.8)
Chest CT changes ≥ 50% GGO—no. (%)	12/24 (50)	6/12 (50)	6/12 (50)
PaO_2_/FiO_2_—median (Q1–Q3)	168 (113–201)	164.1 (106–225)	168.7 (113–223)
Continuous use of immunosuppressants drugs—no. (%)	3 (9)	2 (12.5)	1 (5.6)
**Comorbidities—no. (%)**	27 (79.4)	10 * (62.5)	17 * (94.4)
Systemic arterial hypertension—no. (%)	16 (47)	6 (37.5)	10 (55.5)
*Diabetes mellitus—no.* (%)	12 (35.3)	4 (25)	8 (44.4)
COPD—no. (%)	2 (6)	2 (12.5)	2 (11.1)
Asthma—no. (%)	2 (6)	1 (6.3)	1 (5.6)
Cardiovascular diseases—no. (%)	9 (26.5)	2 (12.5)	7 (38.9)
CKD dialytic—no. (%)	2 (6)	0	2 (11.1)
Active neoplasm—no. (%)	4 (11.8)	1 (6.3)	3 (16.7)
Transplanted—no. (%)	1 (2.9)	1 (6.3)	0
Obesity: BMI > 30 (weight (Kg)/height (m^2^))—no. (%)	7 (20.6)	4(25)	3 (16.7)
Other comorbidities—no. (%)	8 (23.5)	2 (12.5)	6 (33.3)
**Laboratory characteristics at baseline**			
Leukocyte × 10³/µL—median (Q1–Q3)	10.5 (7.1–14)	11.1 (6.7–13.6)	10.4 (6.6–15.1)
Neutrophil × 10³/µL—median (Q1–Q3)	8.7 (6.6–13)	8.6 (5.6–12.2)	8.9 (6.3–14)
Lymphocyte × 10³/µL—median (Q1–Q3)	0.6 (0.4–0.8)	0.7 (0.6–0.8)	0.5 (0.2–0.9)
Platelet × 10³/µL—median (Q1–Q3)	189 (145–450)	199 (155–285)	183 (123–219)
CRP mg/l—median (Q1–Q3)	180 (70–229)	160.2 (57.6–254.4)	190.4 (141.5–228)
Lactate mmol/L—median (Q1–Q3)	1.6 (1.4–4.8)	1.5 (1.2–2.1)	1.7 (1.5–2.2)
Creatinine mg/dL—median (Q1–Q3)	1.6 (0.7–2.2)	1.3 (0.5–6.3)	1.2 (0.6–6.4)
Bilirubin mg/dL—median (Q1–Q3)	0.6 (0.4–5.7)	0.5 (0.3–1.8)	0.6 (0.5–5.7)
**Followup**			
Antibiotic use during the ICU stay—no. (%)	33 (97)	16 (100)	17 (94.4)
Use of dexamethasone during hospitalization—no. (%)	28 (82.4)	14 (87.5)	14 (78)
Vasopressor or inotropic during hospitalization—no. (%)	32 (94.1)	14 (87.5)	18 (100)
AKI during hospitalization—no. (%)	25 (73.5)	10 (62.5)	15 (83.3)
Length of ICU stay in days—median (Q1–Q3)	17 (10.5–33)	21.5 (11–33)	16.5 (9–30)
Length of hospitalization stay in days—median (Q1–Q3)	31 (18.8–44.8)	39 ** (30.5–52.5)	22 ** (13–34)

Abbreviations: AKI, acute kidney injury; APACHE II, Acute Physiology and Chronic Health Evaluation II; BMI, body mass index; COPD, chronic obstructive pulmonary disease; CKD, chronic kidney disease; CRP, C-reactive protein; CT, computed tomography; GGO, ground-glass opacity; ICU, intensive care unit; M, male; PaO_2_/FiO_2_, arterial oxygen partial pressure/fractional inspired oxygen; SOFA, Sequential Organ Failure Assessment. The Mann–Whitney test was used to compare continuous variables, and Fisher’s Exact test for categorical variables. Statistical significance is indicated by * *p* < 0.05 or ** *p* < 0.01.

## Data Availability

The data presented in this study are available on request from the corresponding authors. The data are not publicly available due to restriction regarding confidential information of the patients.

## Supplementary Materials

The following supporting information can be downloaded at: https://www.mdpi.com/article/10.3390/v15081704/s1. Figure S1: Sample collection of survivor and nonsurvivor’ groups; Figure S2: Levels of cytokines in survival and nonsurvivor’ group; Table S1: Correlation matrix between cytokines and chemokines measured in mini-BAL and clinical/laboratory data on days 1–2; Table S2: Correlation matrix between cytokines and chemokines measured in plasma and clinical/laboratory data on days 1–2; Table S3: Correlation matrix between cytokines and chemokines measured in mini-BAL and clinical/laboratory data on days 5–8; Table S4: Correlation matrix between cytokines and chemokines measured in plasma and clinical/laboratory data on days 5–8.

## Abbreviations

ARDS: acute respiratory distress syndrome; COVID-19, coronavirus disease 2019; ICU, intensive care unit; mini-BAL, minibronchoalveolar lavage; MV, mechanical ventilation; PaO_2_/FiO_2_, arterial oxygen partial pressure/fractional inspired oxygen; SARS-CoV-2, severe acute respiratory syndrome coronavirus 2; SOFA, Sequential Organ Failure Assessment; APACHE II, acute physiological assessment and chronic health evaluation.
