# Supplementary material for: Compartmentalized Regulation of Pulmonary and Systemic Inflammation in Critical COVID-19 Patients
Source: Viruses. 2023 Aug 8;15(8):1704. doi: 10.3390/v15081704 (PMC10458965; doi:10.3390/v15081704)
Supplement: Supplementary file 1 [file viruses-15-01704-s001.zip › viruses-2493940-supplementary.pdf]

Supplemental material

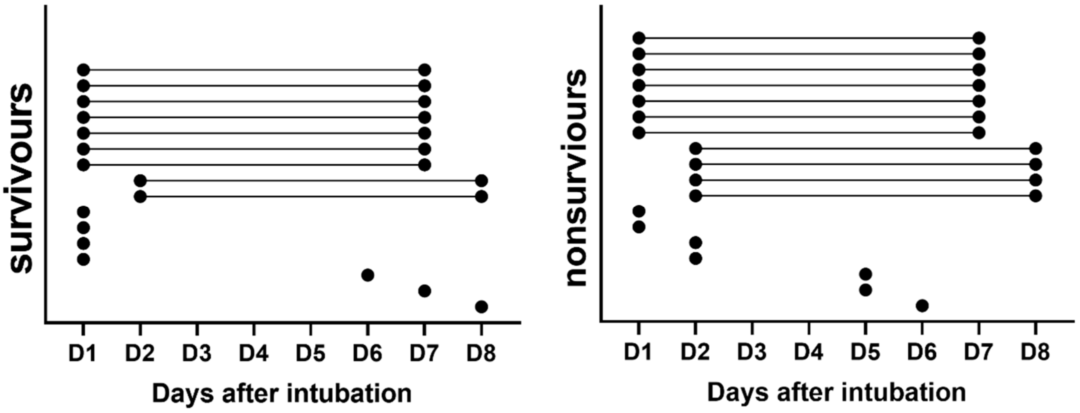

Figure S1: Sample collection of survivors and nonsurvivor` groups. Survivors: *n* = 13 (days 1–2) and *n* = 12 (days 5–8). Non-survivors: *n* = 15 (days 1–2) and *n* = 14 (days 5–8).

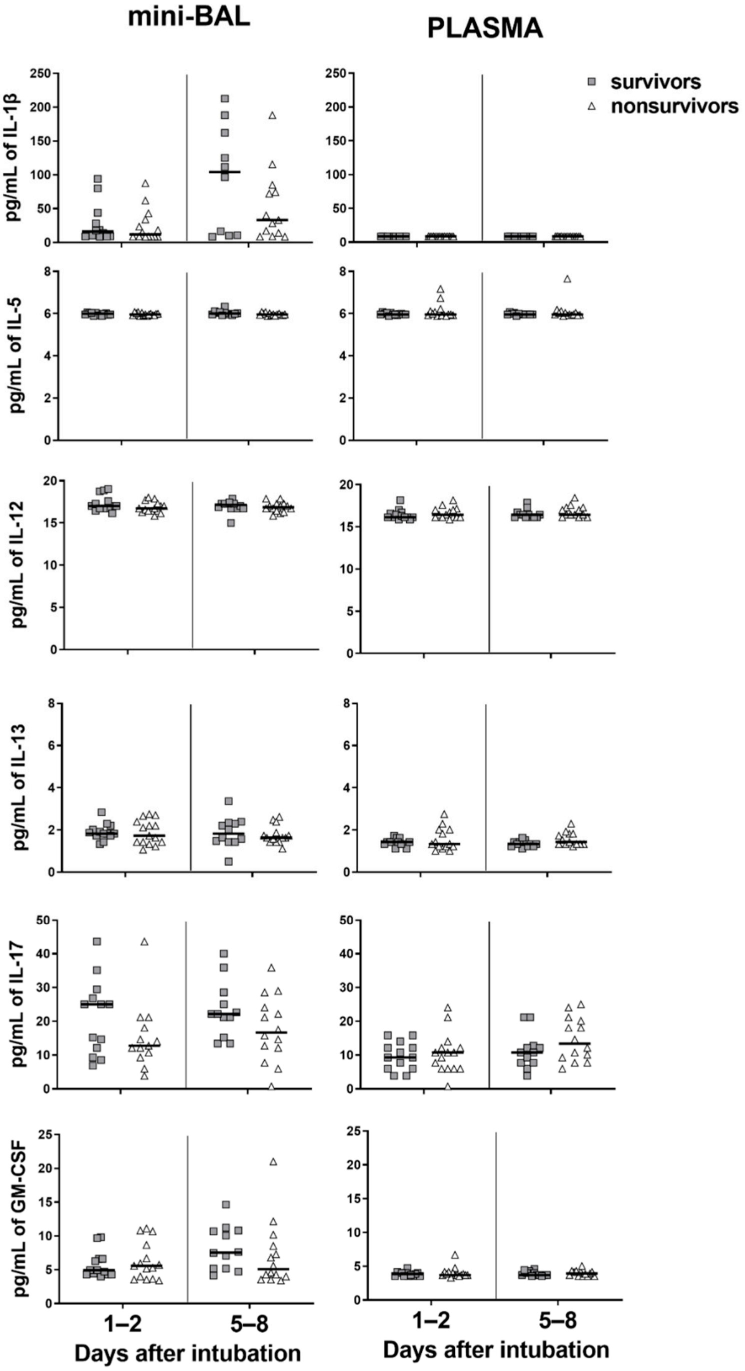

Figure S2: **Levels of cytokines in survival and nonsurvivor' group**. Cytokines measured in mini-BAL and plasma up to 48 hours on the day 1 (days 1─2) and around day 7 (days 5─8) of intubation. Survivors (gray square) *n* = 13 (days1-2) and *n* = 12 (days 5─8). Nonsurvivors (open triangle) *n* = 15 (days 1─2) and *n* = 14 (days 5─8). Differences between groups were analyzed using the Mann-Whitney test. The black lines represent the median of each group. Outliers were identified by the ROUT method (Q=1%) and removed from analysis.

Table S1: Correlation matrix between cytokines and chemokines measured in mini-BAL and clinical/laboratory data on days 1–2.

p/r	CCL2	CCL3	CCL4	CXCL8	CXCL10	TNF	IL-1β	IL-6	IL-12	G-CSF	GM-CSF	IFN-γ	IL-4	IL-5	IL-13	IL-17	IL-2	IL-10	SOFA	CRP	lactate	leukocyte	neutrophil	lymphocyte	platet	creatinine	bilirubin	PaO_2/FiO_2
**CCL2**	1	0.73	0.58	0.46	0.61	0.61	0.32	0.62	0.48	0.64	0.70	0.63	0.67	0.40	0.42	0.63	0.66	0.70	0.28	-0.12	-0.16	-0.07	-0.05	0.16	-0.05	0.11	0.08	-0.02
**CCL3**	0.00	1	0.81	0.55	0.70	0.84	0.46	0.57	0.67	0.71	0.66	0.69	0.77	0.53	0.41	0.75	0.70	0.56	0.11	-0.32	-0.11	-0.21	-0.20	0.09	-0.09	0.19	-0.01	0.17
**CCL4**	0.00	0.00	1	0.73	0.59	0.90	0.73	0.66	0.83	0.83	0.78	0.90	0.87	0.58	0.70	0.90	0.84	0.61	-0.02	-0.41	-0.13	-0.05	-0.07	0.30	0.10	0.11	-0.03	0.23
**CXCL8**	0.01	0.00	0.00	1	0.39	0.83	0.77	0.48	0.71	0.76	0.79	0.70	0.81	0.36	0.54	0.77	0.80	0.50	-0.05	-0.44	-0.02	0.13	0.15	0.49	0.22	0.16	-0.14	0.32
**CXCL10**	0.00	0.00	0.00	0.05	1	0.49	0.13	0.49	0.47	0.36	0.42	0.48	0.51	0.35	0.34	0.45	0.43	0.37	0.38	-0.23	0.01	-0.18	-0.18	0.02	-0.05	-0.10	0.11	-0.12
**TNF**	0.00	0.00	0.00	0.00	0.01	1	0.74	0.61	0.81	0.82	0.78	0.80	0.87	0.56	0.60	0.85	0.85	0.62	-0.04	-0.36	-0.14	-0.05	-0.06	0.42	0.10	0.21	-0.12	0.27
**IL-1β**	0.09	0.01	0.00	0.00	0.51	0.00	1	0.40	0.58	0.65	0.76	0.70	0.73	0.51	0.66	0.67	0.74	0.48	-0.04	-0.40	0.13	0.27	0.21	0.58	0.19	0.30	-0.02	0.34
**IL-6**	0.00	0.00	0.00	0.01	0.01	0.00	0.03	1	0.59	0.69	0.78	0.69	0.69	0.39	0.63	0.75	0.71	0.68	0.02	-0.14	-0.14	-0.08	-0.11	0.29	-0.03	-0.07	0.05	0.02
**IL-12**	0.01	0.00	0.00	0.00	0.01	0.00	0.00	0.00	1	0.76	0.64	0.71	0.73	0.41	0.66	0.86	0.78	0.46	-0.05	-0.38	-0.14	0.01	-0.01	0.44	0.25	0.14	-0.14	0.34
**G-CSF**	0.00	0.00	0.00	0.00	0.07	0.00	0.00	0.00	0.00	1	0.85	0.86	0.91	0.37	0.53	0.90	0.89	0.64	-0.08	-0.41	-0.24	-0.10	-0.11	0.29	-0.07	0.22	-0.15	0.32
**GM-CSF**	0.00	0.00	0.00	0.00	0.03	0.00	0.00	0.00	0.00	0.00	1	0.86	0.90	0.49	0.66	0.85	0.88	0.76	0.07	-0.25	-0.07	0.02	0.00	0.41	0.01	0.17	0.02	0.15
**IFN-γ**	0.00	0.00	0.00	0.00	0.01	0.00	0.00	0.00	0.00	0.00	0.00	1	0.91	0.65	0.70	0.88	0.90	0.74	-0.10	-0.22	-0.12	0.01	-0.01	0.31	-0.02	0.07	0.14	0.17
**IL-4**	0.00	0.00	0.00	0.00	0.01	0.00	0.00	0.00	0.00	0.00	0.00	0.00	1	0.56	0.59	0.88	0.93	0.66	0.00	-0.33	-0.15	-0.01	-0.03	0.31	-0.03	0.14	0.01	0.20
**IL-5**	0.04	0.00	0.00	0.06	0.08	0.00	0.01	0.04	0.03	0.05	0.01	0.00	0.00	1	0.58	0.45	0.60	0.57	-0.10	0.06	0.18	0.11	0.07	0.20	-0.07	0.16	0.43	0.03
**IL-13**	0.02	0.03	0.00	0.00	0.09	0.00	0.00	0.00	0.00	0.00	0.00	0.00	0.00	0.00	1	0.67	0.60	0.69	-0.14	-0.19	0.15	0.12	0.04	0.52	0.14	-0.11	0.31	0.27
**IL-17**	0.00	0.00	0.00	0.00	0.02	0.00	0.00	0.00	0.00	0.00	0.00	0.00	0.00	0.02	0.00	1	0.89	0.66	-0.13	-0.38	-0.17	0.06	0.07	0.34	0.12	0.14	-0.07	0.24
**IL-2**	0.00	0.00	0.00	0.00	0.03	0.00	0.00	0.00	0.00	0.00	0.00	0.00	0.00	0.00	0.00	0.00	1	0.66	0.03	-0.28	-0.13	0.11	0.10	0.39	0.06	0.25	-0.03	0.20
**IL-10**	0.00	0.00	0.00	0.01	0.06	0.00	0.01	0.00	0.01	0.00	0.00	0.00	0.00	0.00	0.00	0.00	0.00	1	0.04	-0.07	-0.02	0.03	-0.03	0.33	-0.10	0.15	0.30	0.07
**SOFA**	0.16	0.57	0.93	0.81	0.05	0.83	0.83	0.90	0.80	0.70	0.72	0.61	0.99	0.61	0.46	0.50	0.88	0.85	1	0.07	0.10	-0.02	-0.09	-0.06	-0.08	0.34	-0.01	-0.23
**CRP**	0.54	0.10	0.03	0.02	0.26	0.06	0.04	0.49	0.05	0.03	0.21	0.26	0.08	0.75	0.34	0.05	0.15	0.72	0.74	1	0.09	-0.08	-0.09	-0.14	-0.22	-0.12	0.41	-0.37
**lactate**	0.43	0.59	0.50	0.92	0.97	0.47	0.50	0.46	0.48	0.21	0.73	0.55	0.44	0.37	0.45	0.38	0.52	0.91	0.61	0.64	1	0.32	0.17	0.40	0.11	-0.03	0.56	-0.06
**leukocyte**	0.74	0.28	0.81	0.50	0.37	0.82	0.16	0.68	0.95	0.61	0.93	0.98	0.95	0.58	0.56	0.74	0.57	0.87	0.94	0.67	0.10	1	0.95	0.41	0.54	0.29	0.12	-0.14
**neutrophil**	0.80	0.30	0.71	0.44	0.39	0.77	0.27	0.58	0.95	0.58	1.00	0.97	0.86	0.74	0.83	0.73	0.62	0.87	0.64	0.66	0.38	0.00	1	0.25	0.54	0.24	0.05	-0.07
**lymphocyte**	0.41	0.66	0.13	0.01	0.94	0.03	0.00	0.13	0.02	0.14	0.03	0.11	0.11	0.32	0.00	0.08	0.04	0.09	0.76	0.49	0.04	0.03	0.20	1	0.31	0.13	0.07	0.08
**platet**	0.79	0.66	0.60	0.26	0.80	0.61	0.33	0.87	0.20	0.71	0.98	0.92	0.89	0.73	0.49	0.56	0.76	0.61	0.70	0.25	0.58	0.00	0.00	0.10	1	-0.15	-0.06	-0.13
**creatinine**	0.57	0.33	0.59	0.43	0.64	0.28	0.13	0.72	0.48	0.26	0.38	0.73	0.48	0.42	0.56	0.48	0.21	0.44	0.08	0.53	0.87	0.14	0.23	0.50	0.43	1	-0.35	0.04
**bilirubin**	0.70	0.96	0.87	0.49	0.58	0.53	0.94	0.81	0.49	0.44	0.91	0.48	0.95	0.02	0.11	0.73	0.86	0.13	0.98	0.03	0.00	0.55	0.80	0.71	0.77	0.07	1	-0.20
**PaO_2/FiO_2**	0.94	0.38	0.23	0.10	0.57	0.16	0.07	0.94	0.08	0.10	0.43	0.40	0.30	0.88	0.17	0.22	0.32	0.72	0.25	0.05	0.77	0.47	0.71	0.69	0.50	0.85	0.31	1

The Spearman rank correlation test was used for statistical analysis. $p < 0.05$ was considered statistically significant.
Abbreviations: CRP, C-reactive protein; G-CSF, granulocyte colony stimulating factor; GM-CSF, granulocyte-macrophage colony-stimulating factor; IFN-γ, interferon γ; IL, interleukin; SOFA, Sequential Organ Failure Assessment; PaO_2/FiO_2, arterial oxygen partial pressure/ fractional inspired oxygen; TNF, tumor necrosis factor.

Table S2: Correlation matrix between cytokines and chemokines measured in plasma and clinical/laboratory data on days 1–2.

p/r	CCL2	CCL3	CCL4	CXCL8	CXCL10	TNF	IL-1β	IL-6	IL-12	G-CSF	GM-CSF	IFN-γ	IL-4	IL-5	IL-13	IL-17	IL-2	IL-10	SOFA	CRP	lactate	leukocyte	neutrophil	lymphocyte	platet	creatinine	bilirubin	PaO_2/FiO_2
**CCL2**	1	0.26	0.27	0.65	0.51	0.53	0.57	0.63	0.51	0.53	0.63	0.71	0.61	0.50	0.73	0.40	0.69	0.44	0.18	0.17	0.13	0.04	0.02	0.03	0.00	-0.01	0.15	-0.37
**CCL3**	0.19	1	0.70	0.42	0.62	0.47	0.50	0.50	0.23	0.63	0.63	0.44	0.53	0.22	0.38	0.59	0.59	0.06	0.12	0.09	0.04	0.29	0.20	0.13	0.27	0.09	0.14	-0.27
**CCL4**	0.17	0.00	1	0.41	0.42	0.40	0.48	0.45	0.17	0.45	0.51	0.48	0.56	0.07	0.36	0.79	0.46	-0.05	-0.14	-0.15	-0.11	0.20	0.15	0.21	0.21	0.09	0.03	-0.13
**CXCL8**	0.00	0.03	0.03	1	0.49	0.61	0.71	0.64	0.47	0.68	0.73	0.66	0.74	0.41	0.47	0.66	0.70	0.26	0.19	-0.02	0.16	0.17	0.01	0.16	-0.09	0.24	0.02	-0.13
**CXCL10**	0.01	0.00	0.03	0.01	1	0.44	0.40	0.67	0.12	0.55	0.47	0.43	0.51	-0.03	0.18	0.33	0.53	0.42	0.14	0.40	0.02	0.03	-0.01	-0.01	0.02	-0.10	0.29	-0.18
**TNF**	0.00	0.01	0.03	0.00	0.02	1	0.81	0.61	0.44	0.80	0.75	0.76	0.80	0.42	0.54	0.65	0.69	0.22	0.13	0.29	-0.01	-0.14	-0.26	-0.12	-0.21	0.21	0.09	-0.35
**IL-1β**	0.00	0.01	0.01	0.00	0.04	0.00	1	0.76	0.54	0.71	0.76	0.77	0.80	0.52	0.56	0.72	0.72	0.30	0.07	0.08	-0.13	0.17	0.05	0.01	0.04	0.31	-0.08	-0.29
**IL-6**	0.00	0.01	0.02	0.00	0.00	0.00	0.00	1	0.41	0.58	0.60	0.74	0.80	0.38	0.45	0.58	0.67	0.37	0.15	0.29	0.00	0.16	0.04	-0.02	0.11	0.04	0.34	-0.39
**IL-12**	0.01	0.24	0.38	0.01	0.54	0.02	0.00	0.03	1	0.40	0.65	0.60	0.56	0.79	0.67	0.48	0.48	0.38	0.04	-0.11	0.04	0.09	0.05	-0.08	0.01	0.23	-0.16	-0.33
**G-CSF**	0.00	0.00	0.02	0.00	0.00	0.00	0.00	0.00	0.04	1	0.80	0.76	0.74	0.37	0.55	0.61	0.87	0.23	0.08	0.20	0.03	0.04	-0.08	0.04	-0.03	0.00	0.17	-0.38
**GM-CSF**	0.00	0.00	0.01	0.00	0.01	0.00	0.00	0.00	0.00	0.00	1	0.81	0.77	0.53	0.60	0.71	0.79	0.18	-0.04	0.13	0.16	0.18	0.05	0.23	0.07	0.10	0.10	-0.36
**IFN-γ**	0.00	0.02	0.01	0.00	0.02	0.00	0.00	0.00	0.00	0.00	0.00	1	0.86	0.57	0.71	0.71	0.76	0.37	0.15	0.15	0.05	0.08	-0.01	0.06	0.04	0.15	0.21	-0.50
**IL-4**	0.00	0.00	0.00	0.00	0.01	0.00	0.00	0.00	0.00	0.00	0.00	0.00	1	0.54	0.61	0.80	0.78	0.32	0.13	0.19	0.01	0.04	-0.08	0.02	-0.07	0.30	0.13	-0.40
**IL-5**	0.01	0.25	0.72	0.03	0.89	0.03	0.00	0.04	0.00	0.06	0.00	0.00	0.00	1	0.81	0.47	0.50	0.31	0.24	-0.05	0.15	0.11	0.08	-0.16	-0.06	0.27	0.03	-0.49
**IL-13**	0.00	0.05	0.06	0.01	0.35	0.00	0.00	0.02	0.00	0.00	0.00	0.00	0.00	0.00	1	0.54	0.64	0.32	0.21	-0.07	0.11	0.09	0.09	-0.09	0.02	0.14	0.05	-0.41
**IL-17**	0.03	0.00	0.00	0.00	0.09	0.00	0.00	0.00	0.01	0.00	0.00	0.00	0.00	0.01	0.00	1	0.61	0.13	-0.06	-0.06	0.10	0.19	0.06	0.19	0.03	0.35	0.03	-0.30
**IL-2**	0.00	0.00	0.01	0.00	0.00	0.00	0.00	0.00	0.01	0.00	0.00	0.00	0.00	0.01	0.00	0.00	1	0.24	0.14	0.11	0.11	0.10	0.00	-0.02	0.14	-0.05	0.21	-0.39
**IL-10**	0.02	0.77	0.79	0.18	0.03	0.25	0.12	0.05	0.05	0.23	0.36	0.05	0.10	0.10	0.10	0.52	0.22	1	0.50	0.10	0.00	-0.15	-0.20	0.06	-0.27	0.26	-0.01	-0.18
**SOFA**	0.37	0.55	0.48	0.34	0.48	0.50	0.73	0.44	0.85	0.70	0.86	0.44	0.50	0.22	0.30	0.78	0.48	0.01	1	0.07	0.10	-0.02	-0.09	-0.06	-0.08	0.34	-0.01	-0.23
**CRP**	0.37	0.65	0.46	0.92	0.04	0.13	0.67	0.13	0.56	0.31	0.50	0.44	0.32	0.80	0.72	0.75	0.58	0.62	0.74	1	0.09	-0.08	-0.09	-0.14	-0.22	-0.12	0.41	-0.37
**lactate**	0.51	0.85	0.57	0.41	0.92	0.95	0.50	0.99	0.82	0.88	0.42	0.81	0.96	0.44	0.58	0.60	0.57	0.98	0.61	0.64	1	0.32	0.17	0.40	0.11	-0.03	0.56	-0.06
**leukocyte**	0.86	0.13	0.31	0.40	0.88	0.49	0.38	0.41	0.64	0.82	0.36	0.67	0.83	0.56	0.63	0.33	0.63	0.43	0.94	0.67	0.10	1	0.95	0.41	0.54	0.29	0.12	-0.14
**neutrophil**	0.92	0.31	0.44	0.95	0.96	0.19	0.79	0.85	0.80	0.70	0.80	0.96	0.69	0.68	0.63	0.76	0.99	0.32	0.64	0.66	0.38	0.00	1	0.25	0.54	0.24	0.05	-0.07
**lymphocyte**	0.89	0.50	0.29	0.41	0.98	0.55	0.98	0.91	0.69	0.86	0.24	0.77	0.93	0.42	0.66	0.33	0.92	0.76	0.76	0.49	0.04	0.03	0.20	1	0.31	0.13	0.07	0.08
**platet**	0.99	0.17	0.27	0.65	0.91	0.28	0.83	0.58	0.97	0.87	0.73	0.82	0.73	0.75	0.94	0.87	0.48	0.17	0.70	0.25	0.58	0.00	0.00	0.10	1	-0.15	-0.06	-0.13
**creatinine**	0.95	0.65	0.66	0.22	0.60	0.27	0.11	0.82	0.23	0.98	0.61	0.46	0.12	0.16	0.48	0.07	0.80	0.18	0.08	0.53	0.87	0.14	0.23	0.50	0.43	1	-0.35	0.04
**bilirubin**	0.43	0.48	0.88	0.90	0.13	0.66	0.70	0.08	0.42	0.38	0.60	0.28	0.50	0.90	0.79	0.89	0.28	0.95	0.98	0.03	0.00	0.55	0.80	0.71	0.77	0.07	1	-0.20
**PaO_2/FiO_2**	0.05	0.17	0.53	0.50	0.37	0.07	0.13	0.04	0.08	0.04	0.06	0.01	0.03	0.01	0.03	0.12	0.04	0.37	0.25	0.05	0.77	0.47	0.71	0.69	0.50	0.85	0.31	1

The Spearman rank correlation test was used for statistical analysis. $p < 0.05$ was considered statistically significant.

Abbreviations: CRP, C-reactive protein; G-CSF, granulocyte colony stimulating factor; GM-CSF, granulocyte-macrophage colony-stimulating factor; IFN-γ, interferon γ; IL, interleukin; SOFA, Sequential Organ Failure Assessment; PaO_2/FiO_2, arterial oxygen partial pressure/ fractional inspired oxygen; TNF, tumor necrosis factor.

Table S3: Correlation matrix between cytokines and chemokines measured in mini-BAL and clinical/laboratory data on days 5–8.

p/r	CCL2	CCL3	CCL4	CXCL8	CXCL10	TNF	IL-1β	IL-6	IL-12	G-CSF	GM-CSF	IFN-γ	IL-4	IL-5	IL-13	IL-17	IL-2	IL-10	SOFA	CRP	lactate	leukocyte	neutrophil	lymphocyte	platet	creatinine	bilirubin	PaO_2/FiO_2
**CCL2**	1	0.67	0.27	0.38	0.45	0.44	0.30	0.49	0.36	0.46	0.73	0.60	0.58	0.34	0.49	0.70	0.58	0.49	-0.19	-0.09	-0.19	0.06	-0.03	-0.20	0.18	0.06	0.04	-0.23
**CCL3**	0.00	1	0.37	0.18	0.44	0.24	0.33	0.35	0.09	0.28	0.41	0.40	0.37	0.02	0.20	0.49	0.40	0.15	-0.26	-0.05	0.04	0.26	0.26	0.01	0.27	0.18	0.03	-0.27
**CCL4**	0.18	0.06	1	0.57	-0.06	0.61	0.33	0.26	0.52	0.26	0.24	0.30	0.36	0.37	0.52	0.49	0.32	0.01	-0.08	-0.13	0.05	-0.27	-0.28	-0.06	-0.05	0.05	-0.10	-0.29
**CXCL8**	0.06	0.37	0.00	1	0.13	0.58	0.27	0.59	0.65	0.43	0.37	0.44	0.42	0.56	0.71	0.62	0.45	0.41	-0.38	-0.29	0.24	-0.34	-0.39	-0.06	0.05	-0.29	0.07	0.03
**CXCL10**	0.02	0.03	0.78	0.52	1	-0.08	0.08	0.36	-0.05	0.36	0.30	0.20	0.13	-0.07	0.04	0.29	0.22	0.13	-0.34	-0.20	0.00	0.27	0.28	-0.03	0.12	0.04	0.24	-0.04
**TNF**	0.02	0.24	0.00	0.00	0.70	1	0.62	0.44	0.79	0.45	0.63	0.70	0.77	0.57	0.82	0.64	0.70	0.42	-0.32	-0.15	0.08	0.00	-0.08	0.12	0.07	-0.10	-0.15	0.06
**IL-1β**	0.14	0.10	0.09	0.18	0.69	0.00	1	0.45	0.37	0.54	0.66	0.75	0.77	0.42	0.43	0.54	0.75	0.41	-0.26	-0.09	0.02	0.35	0.26	0.42	0.27	-0.02	0.15	0.41
**IL-6**	0.01	0.08	0.20	0.00	0.07	0.02	0.02	1	0.42	0.65	0.58	0.75	0.64	0.56	0.47	0.71	0.78	0.61	-0.37	0.04	-0.06	-0.14	-0.20	0.05	0.19	-0.08	0.16	0.17
**IL-12**	0.07	0.68	0.01	0.00	0.80	0.00	0.07	0.03	1	0.30	0.52	0.51	0.62	0.75	0.89	0.58	0.50	0.54	-0.25	-0.11	0.26	-0.14	-0.22	0.03	0.07	-0.39	0.04	-0.21
**G-CSF**	0.02	0.17	0.20	0.03	0.07	0.02	0.00	0.00	0.14	1	0.77	0.75	0.73	0.30	0.30	0.77	0.72	0.32	-0.41	-0.01	-0.08	0.04	-0.05	0.07	0.33	-0.15	0.18	0.22
**GM-CSF**	0.00	0.04	0.25	0.07	0.14	0.00	0.00	0.00	0.01	0.00	1	0.86	0.89	0.46	0.57	0.85	0.84	0.59	-0.24	0.04	-0.09	0.24	0.15	0.07	0.24	-0.14	0.30	0.08
**IFN-γ**	0.00	0.04	0.13	0.03	0.33	0.00	0.00	0.00	0.01	0.00	0.00	1	0.95	0.56	0.57	0.84	0.97	0.60	-0.28	-0.01	0.01	0.12	0.03	0.15	0.37	0.02	0.21	0.28
**IL-4**	0.00	0.06	0.07	0.03	0.54	0.00	0.00	0.00	0.00	0.00	0.00	0.00	1	0.57	0.62	0.84	0.92	0.55	-0.27	-0.07	0.03	0.24	0.13	0.27	0.31	-0.09	0.20	0.24
**IL-5**	0.09	0.93	0.06	0.00	0.72	0.00	0.03	0.00	0.00	0.13	0.02	0.00	0.00	1	0.71	0.56	0.54	0.60	-0.09	-0.02	0.32	-0.16	-0.25	0.05	0.15	-0.31	0.20	0.00
**IL-13**	0.01	0.32	0.01	0.00	0.84	0.00	0.03	0.02	0.00	0.14	0.00	0.00	0.00	0.00	1	0.63	0.57	0.47	-0.19	-0.06	0.17	-0.07	-0.14	-0.01	0.12	-0.25	0.12	-0.12
**IL-17**	0.00	0.01	0.01	0.00	0.15	0.00	0.00	0.00	0.00	0.00	0.00	0.00	0.00	0.00	0.00	1	0.84	0.51	-0.25	-0.04	0.11	0.09	0.02	0.04	0.23	-0.19	0.29	0.08
**IL-2**	0.00	0.05	0.11	0.02	0.28	0.00	0.00	0.00	0.01	0.00	0.00	0.00	0.00	0.00	0.00	0.00	1	0.64	-0.30	0.01	0.01	0.13	0.04	0.20	0.29	0.04	0.18	0.33
**IL-10**	0.01	0.46	0.97	0.04	0.52	0.03	0.04	0.00	0.00	0.11	0.00	0.00	0.00	0.00	0.01	0.01	0.00	1	-0.28	-0.15	0.10	-0.15	-0.21	0.16	0.13	-0.22	0.16	0.05
**SOFA**	0.37	0.21	0.71	0.06	0.10	0.12	0.21	0.07	0.22	0.04	0.26	0.18	0.20	0.67	0.36	0.22	0.14	0.17	1	0.32	-0.13	-0.07	0.00	-0.15	-0.24	0.34	0.34	-0.14
**CRP**	0.66	0.82	0.52	0.14	0.32	0.47	0.67	0.86	0.59	0.96	0.85	0.95	0.74	0.92	0.75	0.86	0.98	0.45	0.12	1	-0.41	0.10	0.09	-0.22	0.13	0.28	-0.11	-0.13
**lactate**	0.36	0.86	0.80	0.24	0.99	0.71	0.93	0.79	0.20	0.71	0.66	0.96	0.87	0.11	0.40	0.61	0.97	0.64	0.52	0.04	1	0.10	0.15	0.16	0.04	-0.43	0.39	0.11
**leukocyte**	0.77	0.19	0.18	0.09	0.18	0.98	0.08	0.51	0.48	0.86	0.24	0.55	0.25	0.42	0.74	0.66	0.51	0.48	0.75	0.64	0.63	1	0.97	0.54	0.17	0.05	0.13	0.29
**neutrophil**	0.89	0.19	0.16	0.05	0.17	0.70	0.20	0.34	0.28	0.79	0.47	0.87	0.52	0.22	0.51	0.94	0.83	0.31	1.00	0.68	0.48	0.00	1	0.49	0.11	0.07	0.22	0.25
**lymphocyte**	0.34	0.98	0.76	0.77	0.89	0.57	0.03	0.82	0.87	0.75	0.74	0.47	0.18	0.80	0.95	0.85	0.32	0.43	0.48	0.27	0.43	0.00	0.01	1	0.20	0.05	-0.04	0.34
**platet**	0.37	0.18	0.80	0.80	0.56	0.73	0.18	0.36	0.72	0.09	0.24	0.06	0.12	0.47	0.55	0.26	0.15	0.53	0.24	0.53	0.84	0.42	0.58	0.33	1	0.24	-0.28	0.08
**creatinine**	0.76	0.37	0.82	0.15	0.83	0.61	0.93	0.71	0.05	0.46	0.49	0.92	0.68	0.12	0.21	0.35	0.86	0.28	0.09	0.16	0.03	0.80	0.73	0.82	0.24	1	-0.45	0.04
**bilirubin**	0.85	0.90	0.67	0.76	0.31	0.54	0.51	0.49	0.85	0.45	0.19	0.37	0.40	0.41	0.62	0.22	0.45	0.51	0.15	0.64	0.09	0.57	0.36	0.88	0.24	0.05	1	0.09
**PaO_2/FiO_2**	0.26	0.18	0.16	0.88	0.83	0.78	0.04	0.42	0.29	0.27	0.71	0.16	0.24	0.99	0.55	0.71	0.10	0.82	0.50	0.53	0.60	0.15	0.22	0.09	0.69	0.84	0.72	1

The Spearman rank correlation test was used for statistical analysis. $p < 0.05$ was considered statistically significant.
Abbreviations: CRP, C-reactive protein; G-CSF, granulocyte colony stimulating factor; GM-CSF, granulocyte-macrophage colony-stimulating factor; IFN-γ, interferon γ; IL, interleukin; SOFA, Sequential Organ Failure Assessment; PaO_2/FiO_2, arterial oxygen partial pressure/ fractional inspired oxygen; TNF, tumor necrosis factor.

Table S4: Correlation matrix between cytokines and chemokines measured in plasma and clinical/laboratory data on days 5–8.

p/r	CCL2	CCL3	CCL4	CXCL8	CXCL10	TNF	IL-1β	IL-6	IL-12	G-CSF	GM-CSF	IFN-γ	IL-4	IL-5	IL-13	IL-17	IL-2	IL-10	SOFA	CRP	lactate	leukocyte	neutrophil	lymphocyte	platet	creatinine	bilirubin	PaO_2/FiO_2
**CCL2**	1	0.27	0.42	0.41	0.42	0.61	0.76	0.62	0.73	0.56	0.55	0.59	0.65	0.33	0.74	0.52	0.70	0.63	0.09	0.29	0.07	0.01	0.07	-0.19	0.12	0.16	0.09	-0.17
**CCL3**	0.18	1	0.60	0.58	0.76	0.53	0.49	0.27	0.25	0.28	0.28	0.23	0.42	0.26	0.24	0.55	0.39	0.18	0.11	-0.16	0.10	0.05	0.03	-0.05	0.08	0.23	0.11	0.40
**CCL4**	0.03	0.00	1	0.59	0.59	0.48	0.44	0.47	0.40	0.47	0.45	0.18	0.31	0.20	0.46	0.88	0.47	0.40	0.44	0.06	0.09	-0.07	0.05	-0.21	-0.22	0.35	0.22	0.18
**CXCL8**	0.04	0.00	0.00	1	0.40	0.50	0.58	0.49	0.35	0.53	0.52	0.42	0.43	0.24	0.33	0.57	0.50	0.36	0.24	0.20	-0.17	0.07	0.09	-0.33	-0.31	0.29	0.07	0.26
**CXCL10**	0.03	0.00	0.00	0.04	1	0.62	0.54	0.24	0.47	0.41	0.31	0.40	0.52	0.40	0.36	0.55	0.45	0.54	0.24	-0.19	0.17	-0.07	-0.03	-0.06	-0.11	0.15	0.29	0.12
**TNF**	0.00	0.01	0.01	0.01	0.00	1	0.80	0.47	0.65	0.68	0.67	0.71	0.74	0.60	0.52	0.65	0.80	0.47	0.10	0.16	-0.16	0.05	0.01	-0.05	0.05	0.22	0.00	0.04
**IL-1β**	0.00	0.01	0.02	0.00	0.00	0.00	1	0.55	0.72	0.80	0.82	0.65	0.77	0.37	0.54	0.63	0.64	0.50	0.13	0.37	-0.14	0.08	0.08	-0.15	0.09	0.33	-0.06	-0.01
**IL-6**	0.00	0.18	0.02	0.01	0.24	0.02	0.00	1	0.38	0.64	0.60	0.43	0.42	0.28	0.52	0.59	0.47	0.21	0.23	0.47	0.09	0.22	0.22	-0.11	0.10	0.20	0.19	0.23
**IL-12**	0.00	0.22	0.04	0.08	0.02	0.00	0.00	0.05	1	0.59	0.62	0.69	0.61	0.52	0.71	0.54	0.72	0.63	0.20	0.09	-0.14	-0.13	-0.08	-0.19	-0.06	0.12	0.08	-0.27
**G-CSF**	0.00	0.17	0.01	0.01	0.04	0.00	0.00	0.00	0.00	1	0.91	0.63	0.55	0.30	0.59	0.59	0.53	0.42	0.14	0.35	-0.12	0.01	0.01	-0.24	-0.04	0.40	-0.16	-0.18
**GM-CSF**	0.00	0.16	0.02	0.01	0.13	0.00	0.00	0.00	0.00	0.00	1	0.56	0.59	0.25	0.52	0.60	0.55	0.32	0.04	0.37	-0.24	-0.02	-0.03	-0.15	0.02	0.40	-0.34	0.00
**IFN-γ**	0.00	0.26	0.37	0.03	0.04	0.00	0.00	0.03	0.00	0.00	0.00	1	0.60	0.47	0.53	0.35	0.80	0.54	-0.14	-0.02	-0.02	0.23	0.19	-0.04	-0.17	-0.04	0.12	-0.11
**IL-4**	0.00	0.03	0.12	0.03	0.01	0.00	0.00	0.03	0.00	0.00	0.00	0.00	1	0.30	0.36	0.40	0.68	0.55	-0.13	0.17	0.06	0.11	0.13	0.06	0.17	0.03	0.07	0.12
**IL-5**	0.09	0.20	0.32	0.23	0.04	0.00	0.06	0.16	0.01	0.13	0.22	0.02	0.14	1	0.35	0.39	0.50	0.19	0.13	0.17	-0.19	-0.19	-0.26	-0.40	0.01	-0.10	0.22	-0.09
**IL-13**	0.00	0.24	0.02	0.10	0.07	0.01	0.00	0.01	0.00	0.00	0.01	0.01	0.07	0.08	1	0.52	0.63	0.51	0.28	0.02	0.12	-0.27	-0.24	-0.21	-0.09	0.11	0.03	-0.35
**IL-17**	0.01	0.00	0.00	0.00	0.00	0.00	0.00	0.00	0.00	0.00	0.00	0.08	0.04	0.05	0.01	1	0.55	0.38	0.48	0.31	-0.07	-0.04	0.02	-0.17	-0.20	0.41	0.23	0.15
**IL-2**	0.00	0.05	0.02	0.01	0.02	0.00	0.00	0.01	0.00	0.00	0.00	0.00	0.00	0.01	0.00	0.00	1	0.59	-0.12	0.05	-0.09	0.09	0.10	-0.04	-0.12	0.00	-0.02	-0.02
**IL-10**	0.00	0.37	0.04	0.07	0.00	0.02	0.01	0.29	0.00	0.03	0.11	0.00	0.00	0.35	0.01	0.05	0.00	1	0.26	0.04	-0.13	0.02	0.13	-0.02	-0.20	0.24	0.19	-0.23
**SOFA**	0.66	0.59	0.03	0.25	0.25	0.64	0.54	0.26	0.34	0.51	0.83	0.51	0.53	0.55	0.18	0.01	0.57	0.21	1	0.32	-0.13	-0.07	0.00	-0.15	-0.24	0.34	0.34	-0.14
**CRP**	0.15	0.44	0.75	0.32	0.35	0.42	0.07	0.02	0.65	0.08	0.06	0.91	0.41	0.42	0.91	0.13	0.81	0.86	0.12	1	-0.41	0.10	0.09	-0.22	0.13	0.28	-0.11	-0.13
**lactate**	0.74	0.63	0.65	0.40	0.40	0.43	0.51	0.67	0.49	0.57	0.25	0.93	0.77	0.36	0.55	0.74	0.67	0.53	0.52	0.04	1	0.10	0.15	0.16	0.04	-0.43	0.39	0.11
**leukocyte**	0.97	0.80	0.72	0.72	0.74	0.82	0.71	0.27	0.52	0.98	0.91	0.26	0.59	0.36	0.18	0.84	0.65	0.91	0.75	0.64	0.63	1	0.97	0.54	0.17	0.05	0.13	0.29
**neutrophil**	0.75	0.89	0.79	0.65	0.87	0.97	0.69	0.29	0.71	0.97	0.90	0.36	0.53	0.20	0.23	0.93	0.63	0.51	1.00	0.68	0.48	0.00	1	0.49	0.11	0.07	0.22	0.25
**lymphocyte**	0.35	0.82	0.29	0.10	0.78	0.82	0.45	0.60	0.35	0.23	0.45	0.86	0.78	0.05	0.30	0.41	0.85	0.93	0.48	0.27	0.43	0.00	0.01	1	0.20	0.05	-0.04	0.34
**platet**	0.56	0.70	0.29	0.12	0.59	0.81	0.67	0.62	0.77	0.85	0.92	0.40	0.40	0.98	0.68	0.33	0.55	0.32	0.24	0.53	0.84	0.42	0.58	0.33	1	0.24	-0.28	0.08
**creatinine**	0.43	0.27	0.08	0.15	0.45	0.29	0.10	0.32	0.57	0.04	0.04	0.84	0.89	0.63	0.58	0.04	1.00	0.23	0.09	0.16	0.03	0.80	0.73	0.82	0.24	1	-0.45	0.04
**bilirubin**	0.71	0.63	0.35	0.76	0.22	0.99	0.81	0.42	0.75	0.49	0.15	0.60	0.78	0.35	0.89	0.34	0.92	0.42	0.15	0.64	0.09	0.57	0.36	0.88	0.24	0.05	1	0.09
**PaO_2/FiO_2**	0.42	0.04	0.39	0.20	0.57	0.83	0.95	0.25	0.18	0.38	1.00	0.59	0.55	0.67	0.08	0.48	0.93	0.27	0.50	0.53	0.60	0.15	0.22	0.09	0.69	0.84	0.72	1

The Spearman rank correlation test was used for statistical analysis. $p < 0.05$ was considered statistically significant.

Abbreviations: CRP, C-reactive protein; G-CSF, granulocyte colony stimulating factor; GM-CSF, granulocyte-macrophage colony-stimulating factor; IFN-γ, interferon γ; IL, interleukin; SOFA, Sequential Organ Failure Assessment; PaO_2/FiO_2, arterial oxygen partial pressure/ fractional inspired oxygen; TNF, tumor necrosis factor.
